# Supplementary material for: Consummating ion desolvation in hard carbon anodes for reversible sodium storage
Source: Nat Commun. 2024 Apr 25;15:3497. doi: 10.1038/s41467-024-47522-y (PMC11045730; doi:10.1038/s41467-024-47522-y)
Supplement: Supplementary file 1 — Supplementary Information [file 41467_2024_47522_MOESM1_ESM.pdf]

Supplementary Materials for

Consummating ion desolvation in hard carbon anodes for reversible sodium storage

Ziyang Lu[1#], Huijun Yang[1#], Yong Guo[2], Hongxin Lin[3], Peizhao Shan[3], Shichao Wu[2], Ping He[4], Yong Yang[3], Quan-Hong Yang[2]* and Haoshen Zhou[1,4]*

[1]Graduate School of System and Information Engineering, University of Tsukuba, 1-1-1, Tennoudai, Tsukuba 305-8573, Japan.

[2]Nanoyang Group, Tianjin Key Laboratory of Advanced Carbon and Electrochemical Energy Storage, School of Chemical Engineering and Technology, and Collaborative Innovation Center of Chemical Science and Engineering (Tianjin), Tianjin University, Tianjin 300072, P. R. China.

[3]State Key Laboratory for Physical Chemistry of Solid Surfaces, Collaborative Innovation Center of Chemistry for Energy Materials and Department of Chemistry, College of Chemistry and Chemical Engineering, Xiamen University, Xiamen, P. R. China.

[4]Center of Energy Storage Materials & Technology, College of Engineering and Applied Sciences, Jiangsu Key Laboratory of Artificial Functional Materials, National Laboratory of Solid State Micro-structures, and Collaborative Innovation Center of Advanced Micro-structures, Nanjing University, Nanjing 210093, P. R. China.

[#]These authors contributed equally: Ziyang Lu, Huijun Yang.

The present affiliation for Prof. Haoshen Zhou is Nanjing University.

*Corresponding author. Email: qhyangcn@tju.edu.cn; hszhou@nju.edu.cn (H. Z.)

Inventory of Supporting Information

- Supplementary Figures S1 to S29
- Supplementary Tables S1 to S4
- Supplementary References

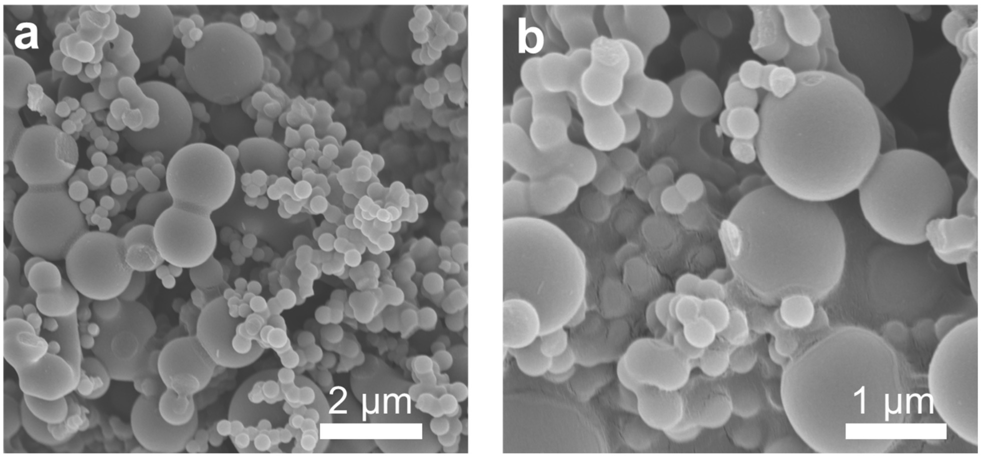

Supplementary Figure 1 | SEM images of hard carbon used in this work. The SEM image of prepared hard carbon anode (a) and the SEM image (b) with higher resolution.

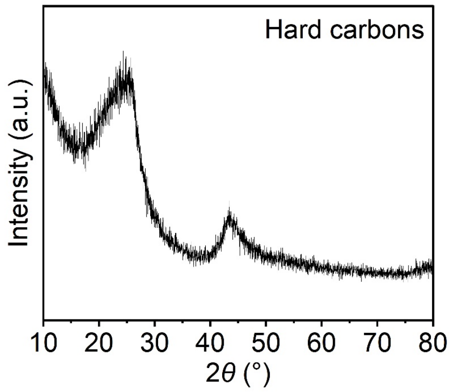

Supplementary Figure 2 | XRD pattern of the hard carbon used in this work.

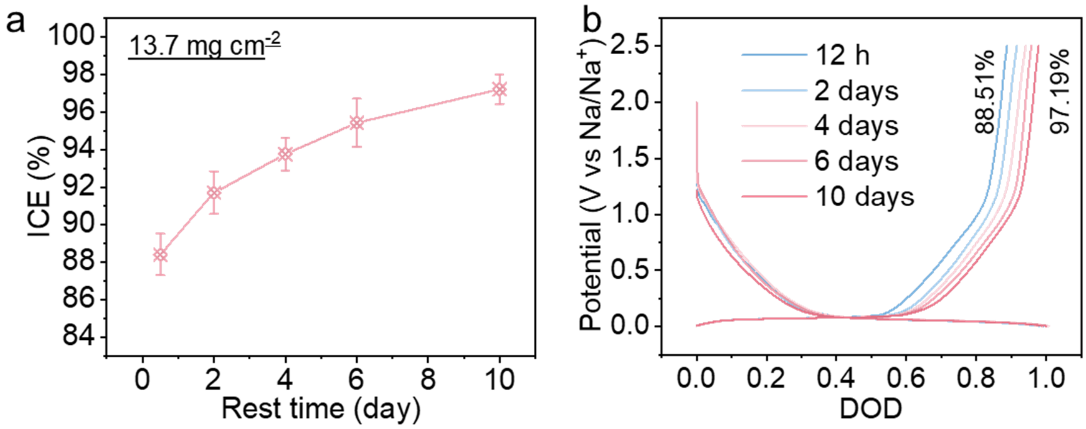

Supplementary Figure 3 | a, The curve of ICE versus resting time for hard carbons with high mass loading. b, The initial charge-discharge curve at different resting time. The test was conducted at specific current of 20 mA g^{-1}.

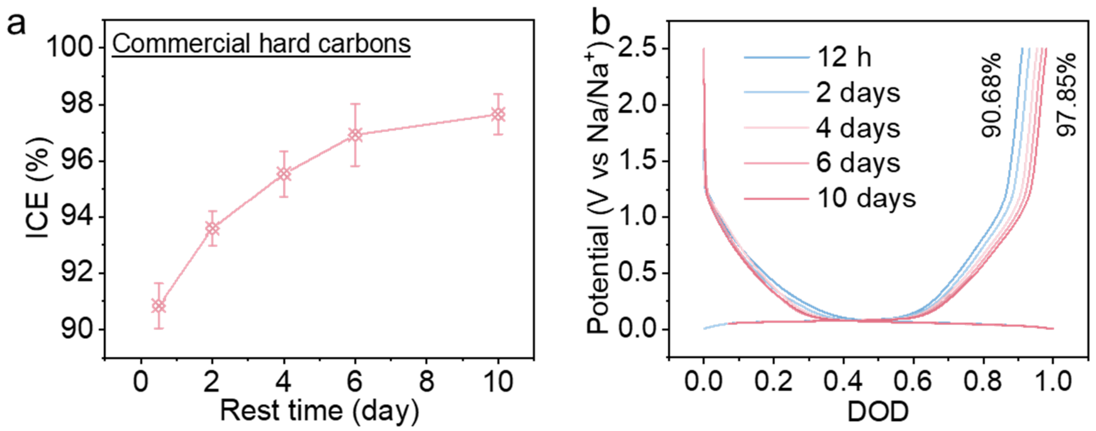

Supplementary Figure 4 | a, The curve of ICE versus resting time for commercial hard carbons. b, The initial charge-discharge curve at different resting time. The test was conducted at specific current of 20 mA g^{-1}.

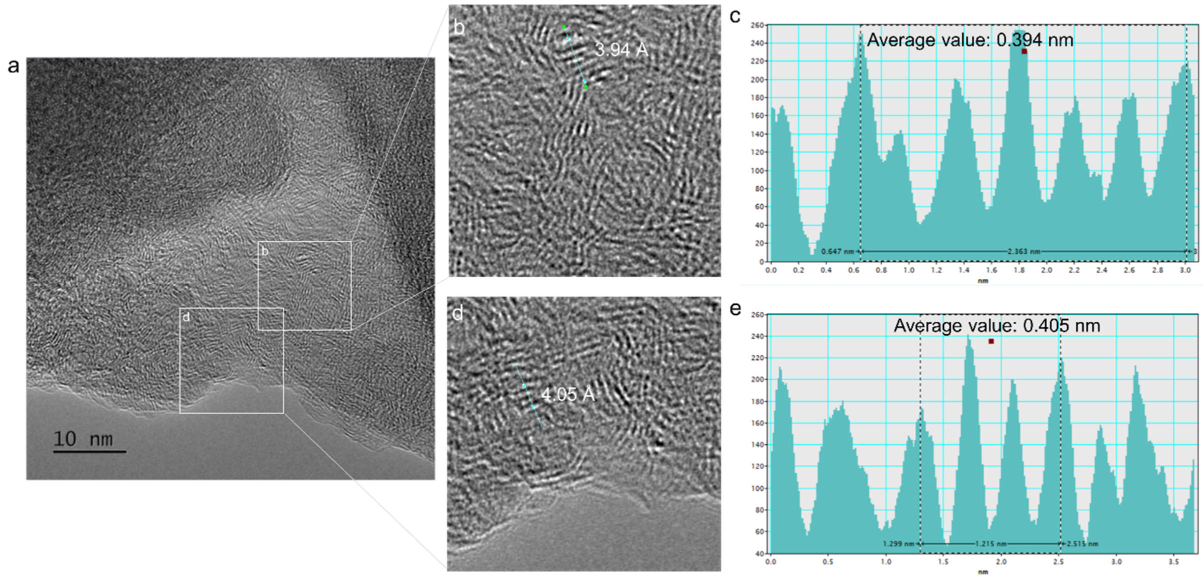

Supplementary Figure 5 | TEM analysis of pristine hard carbons. (a) TEM image of pristine hard carbon. (b) enlarged region cycled in Figure a. (c) Corresponding intensity profiles obtained the line in Figure b. (d) enlarged region cycled in Figure a. (e) Corresponding intensity profiles obtained the line in Figure d.

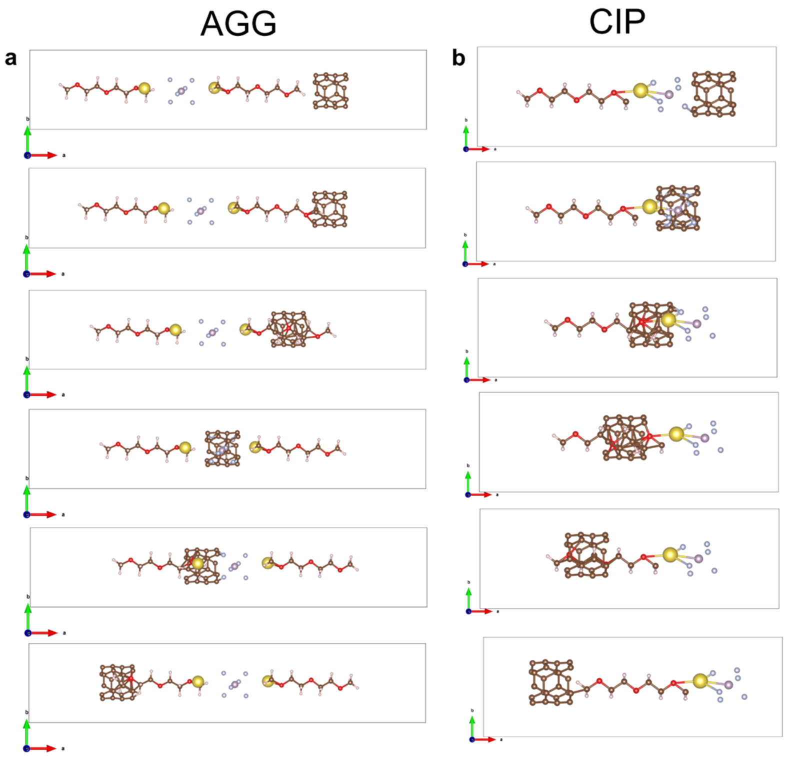

Supplementary Figure 6 | Stable transition state structure. The intermediate state of the AGG (a) and CIP (b) solvation structure when passing through the nanopore of hard carbons.

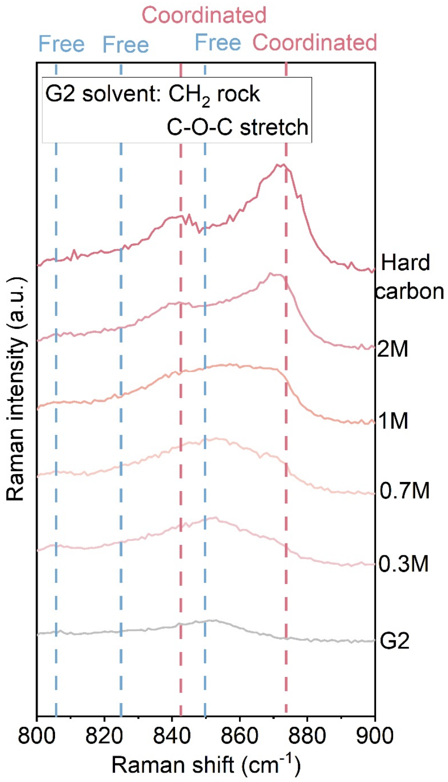

Supplementary Figure 7 | SEM images of hard carbon used in this work. Raman spectroscopy of $NaPF_6$-G2 electrolytes with different concentrations and electrolyte configuration in the nanopore of hard carbons.

Three peaks can be observed for pure G2 solvent, corresponding to the C-O-C stretching and CH_2 rocking of free solvents. When increasing the concentration, two additional peaks of coordinated solvent appear and undergo a slight blue-shift due to the ion-dipole attraction between dissociated Na^+ and oxygens of solvents. For the electrolytes sieved by hard carbon, it shows two main peaks corresponding to coordinated solvent, which is similar to the saturated $NaPF_6$-G2 electrolyte.

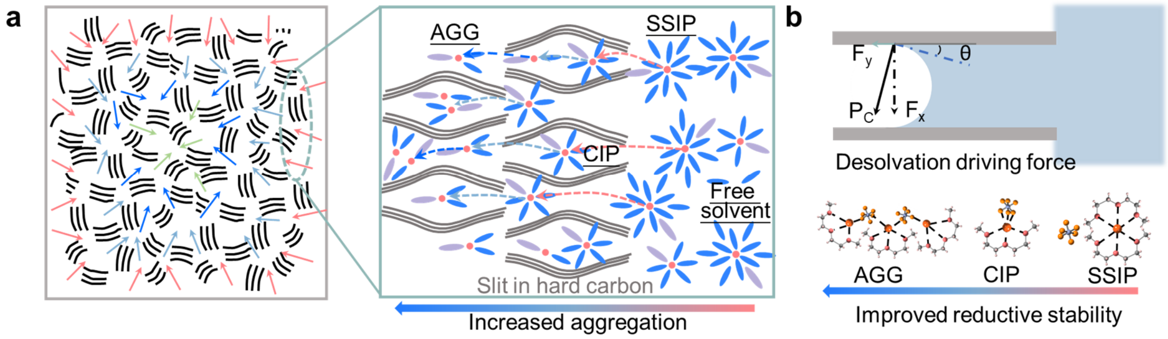

Supplementary Figure 8 | Schematic diagram of electrolyte pre-desolvation on the hard carbons and pre-desolvation driving force diagram. (a) The evolution of the solvation structure of liquid electrolyte in hard carbon anodes. (b) The desolvation process driven from capillary and the comparison of reductive stability of different solvation structures.

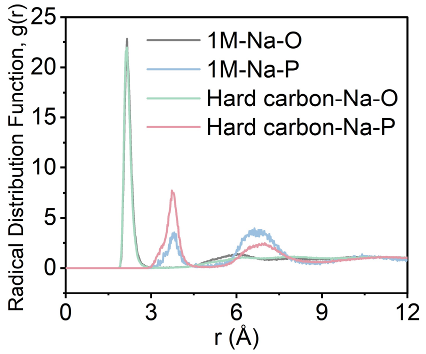

Supplementary Figure 9 | Na^+ radial distribution function of 1M $NaPF_6$-G2 electrolytes and the electrolyte in hard carbons acquired from MD simulations.

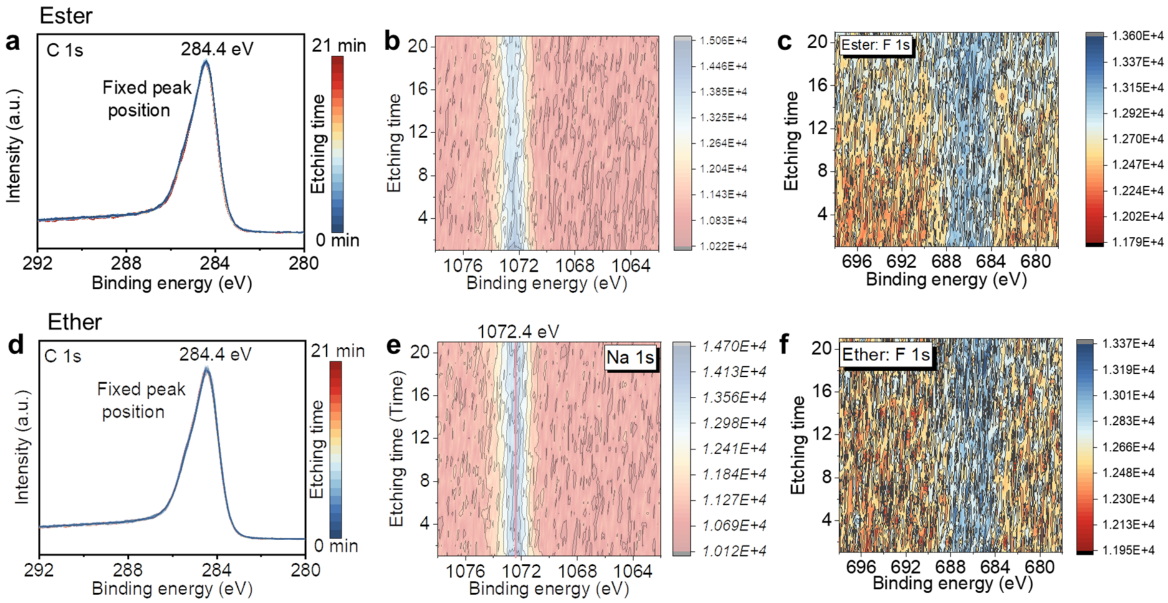

Supplementary Figure 10 | XPS spectra of hard carbons immersed in electrolytes. The C 1*s* (a), Na 1*s* (b) and F 1*s* (c) spectra with different etching time (0-21 min) collected from the hard carbon immersing in 1 M $NaPF_6$-EC/DEC electrolytes. The C 1*s* (d), Na 1*s* (e) and F 1*s* (f) spectra with different etching time (0-21 min) collected from the hard carbon immersing in 1 M $NaPF_6$-G2 electrolytes (without Na metal).

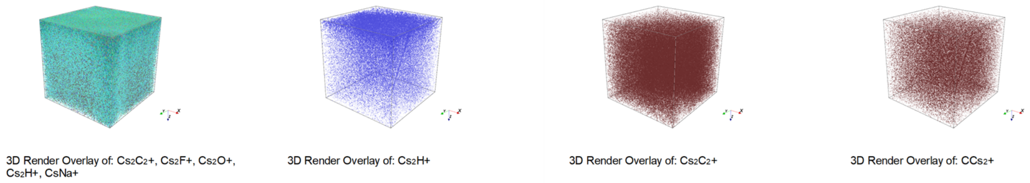

Supplementary Figure 11 | TOF-SIMS results. Positive TOF-SIMS mapping of hard carbons after immersing in 1 M $NaPF_6$-G2 electrolytes (without Na metal) for one week.

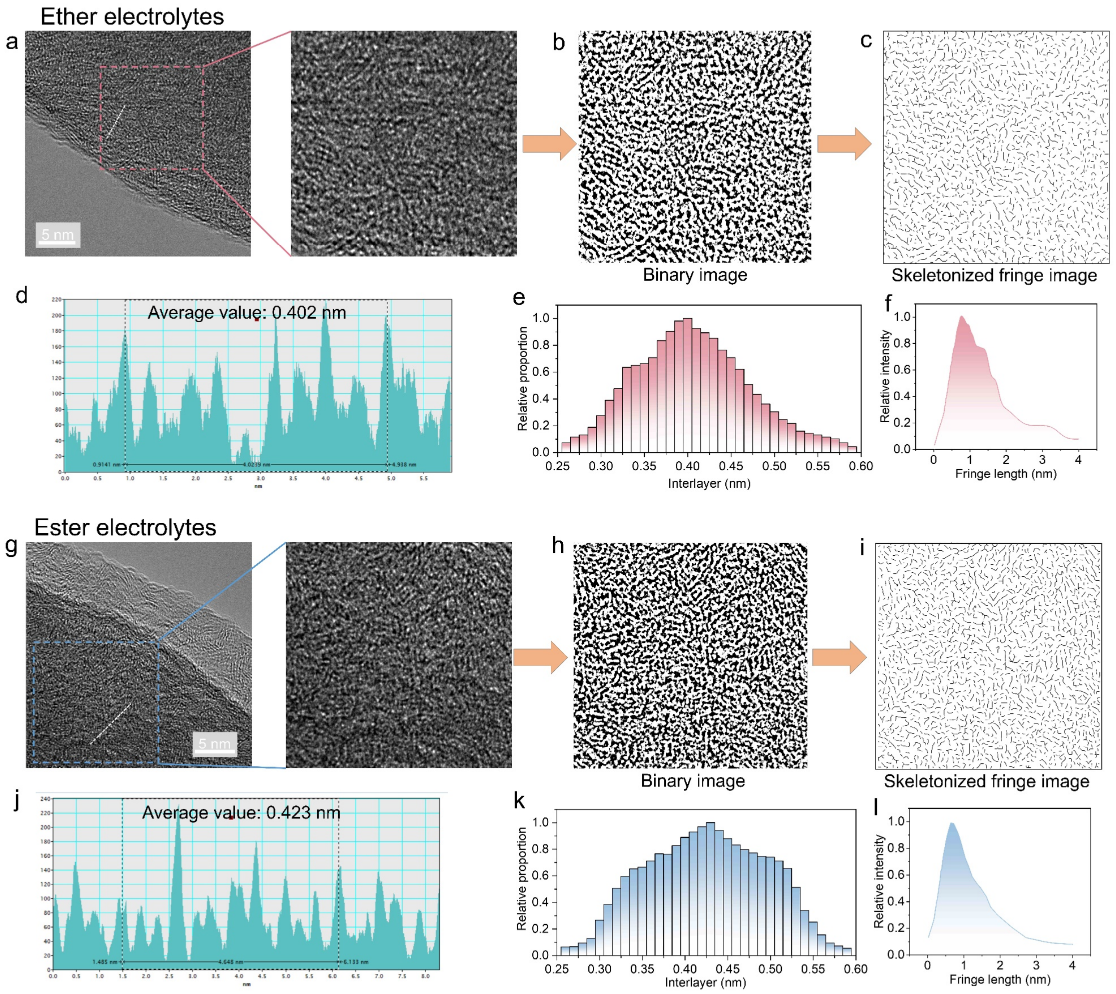

Supplementary Figure 12 | TEM analysis of hard carbons cycled in different electrolytes. (a) The HRTEM image of hard carbon harvested from ether electrolytes and corresponding preprocessed image. (b) Corresponding binary image. (c) The skeletonized fringe image. (d) The intensity profiles obtained from the line in Figure a. (e) Interlayer spacing distribution statistics for Figure c. (f) Fringe length distribution statistics for Figure c. (g) The HRTEM image of hard carbon harvested from ester electrolytes and corresponding preprocessed image. (h) Corresponding binary image. (i) The skeletonized fringe image. (j) The intensity profiles obtained from the line in Figure a. (k) Interlayer spacing distribution statistics for Figure c. (l) Fringe length distribution statistics for Figure i.

In order to obtain more accurate and fine structural information from HRTEM images, the intelligent fringe recognition method was applied to identify structural characteristics of hard carbons according to previous studies[10-12]. The resulting binary image after the filtering shows clearer fringe compared to the original HRTEM image. Finally, the skeletonized fringe image can be obtained after branch pruning, which was used for data statistics and analysis. For the case cycled in ether electrolytes, the interlayer spacing is mainly concentrated around 0.4 nm (Supplementary Figure 12e), which is much smaller than the case using ester electrolytes. For the fringe length, it is mainly distributed around 0.77 nm, and there is a strong peak near 1.5 nm, which is longer than the hard carbon harvested from ester electrolytes. The increased interlayer spacing and reduced fringe length can be attributed to the irreversible intercalation of Na^+ in hard carbon, which expands the graphene sheets and interrupts the original continuous layered structure.

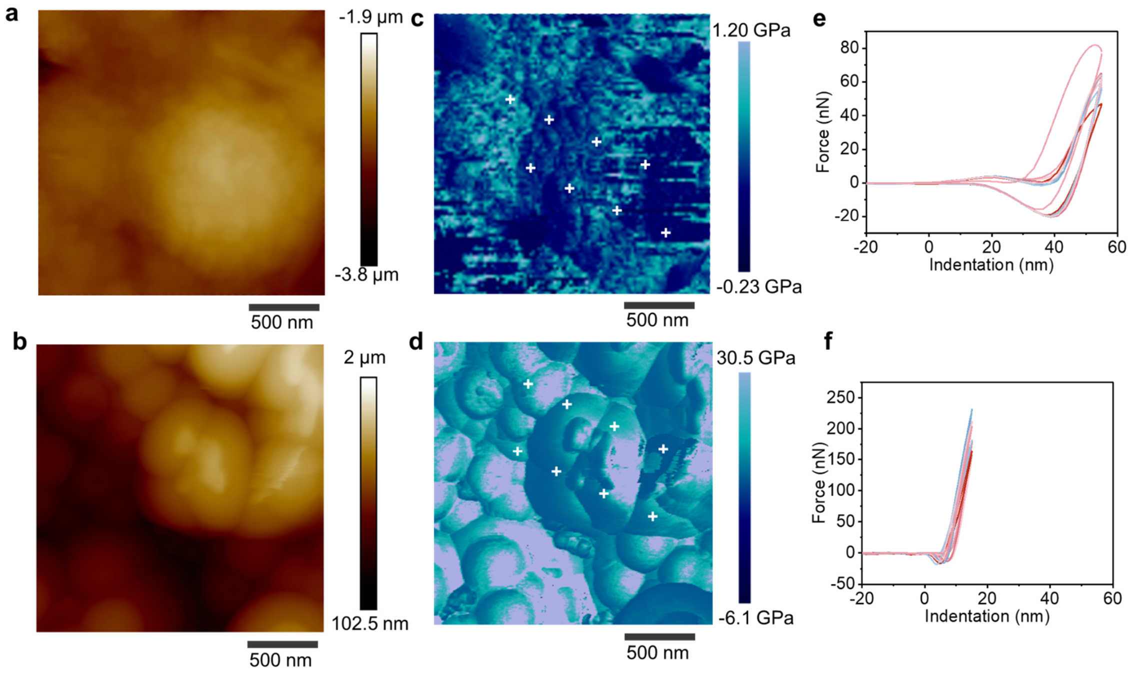

Supplementary Figure 13 | AFM analysis of SEI formed in those two electrolytes. AFM height images of cycled hard carbon in 1M $NaPF_6$-EC/DEC electrolytes (a) and 1M $NaPF_6$-G2 electrolytes (b). Two-dimensional AFM maps of elastic modulus AFM analysis of cycled hard carbons in 1 M $NaPF_6$-EC/DEC electrolytes (c) and 1M $NaPF_6$-G2 electrolytes (d). e, f, Corresponding representative force-displacement curves of selected sites in Fig. c and Fig. d.

The force displacement curves indicate that the SEI formed in ester electrolytes has a good flexibility as the loading and unloading curves are not fully irreversible, which is typical characteristic of organics. But for the SEI formed in ether electrolytes, it shows small displacement in elastic deformation, which is closer to the mechanical properties of pristine hard carbons.

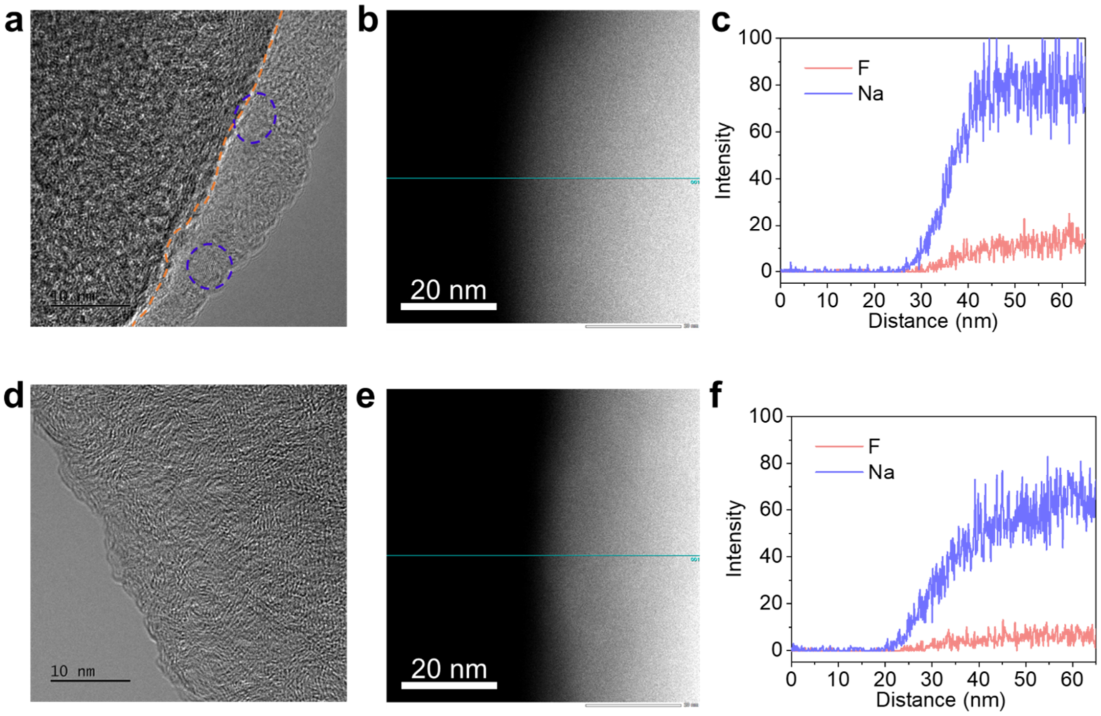

Supplementary Figure 14 | TEM analysis of cycled hard carbons. a, TEM image of cycled hard carbon harvested from ester electrolytes. HAADF images of the hard carbon (b) and corresponding element distribution of Na and F (c). d, TEM image of cycled hard carbon harvested from ether electrolytes. HAADF images of the hard carbon (e) and corresponding element distribution of Na and F (f).

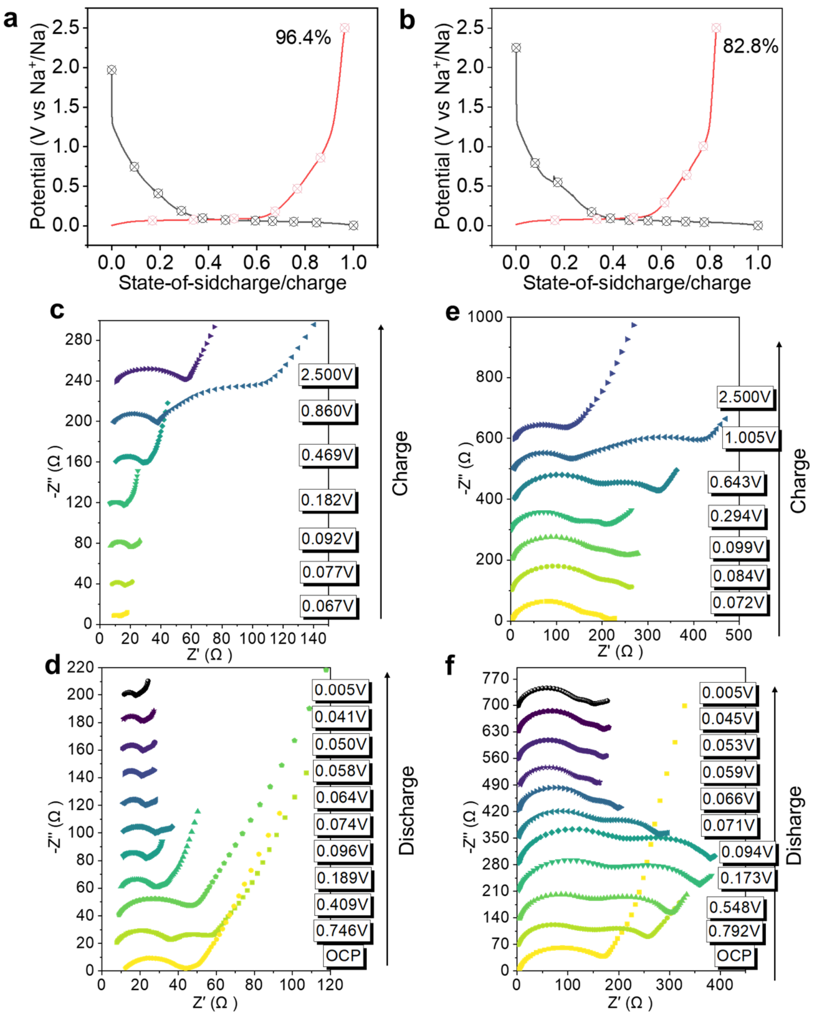

Supplementary Figure 15 | In-situ EIS tests of hard carbon anode in ester and ether electrolytes. The initial charge-discharge curve during EIS measurement in ester (a) and ether (b) electrolytes. The EIS of hard carbon anodes at different discharge-charge state in ether (c-d) and ester (e-f) electrolytes. The test was conducted at specific current of 20 mA g^{-1}.

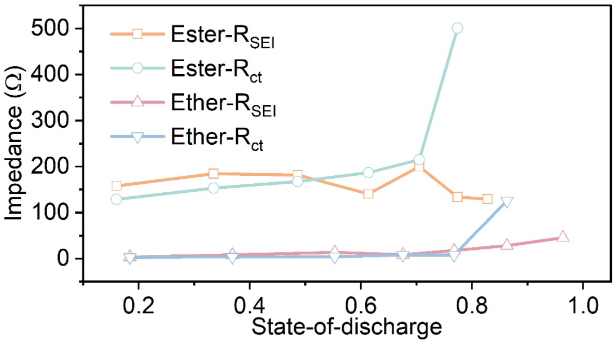

Supplementary Figure 16 | Impedance changes during charging. The Na^+ transport resistance through SEI and charge transfer resistance (R_{ct}) at different charge state in ester electrolytes and ether electrolytes.

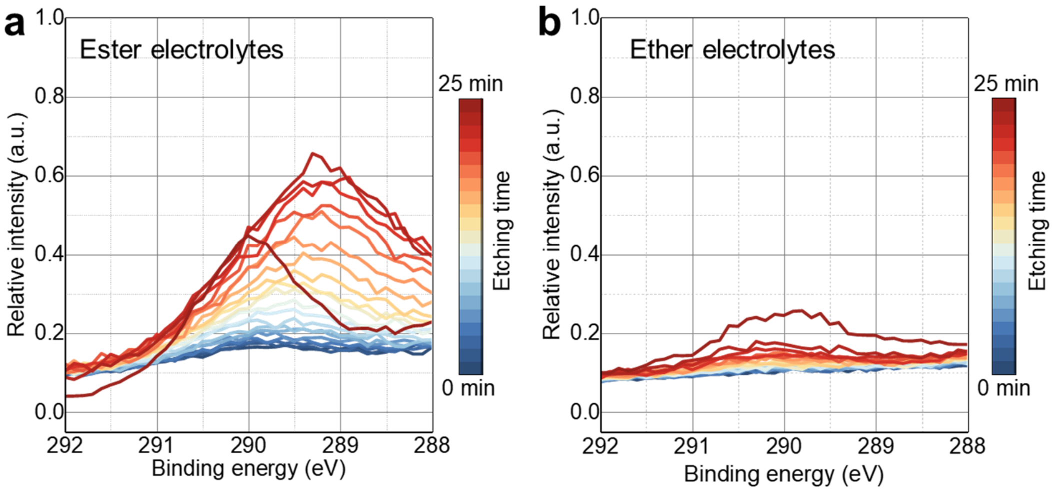

Supplementary Figure 17 | Changes of organic composition with etching. Organic composition of C 1s with etching time from 0-25 min collected from the cycled hard carbons in 1 M $NaPF_6$-EC/DEC electrolytes (a) and 1 M $NaPF_6$-G2 electrolytes (b).

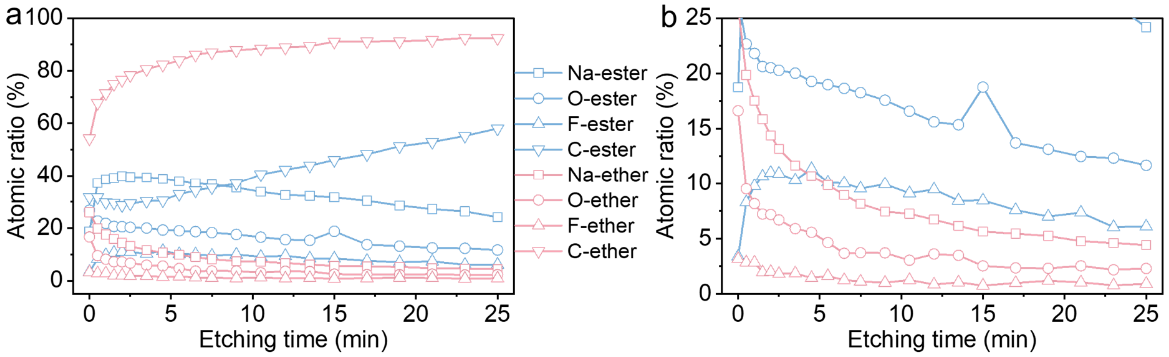

Supplementary Figure 18 | Comparison of element content in SEI formed in different electrolytes. a, XPS atomic concentration of various elements as a function of sputtering time for the SEI formed in 1 M $NaPF_6$-EC/DEC electrolytes and 1 M $NaPF_6$-G2 electrolytes. b, Corresponding enlarged region from 0-25%.

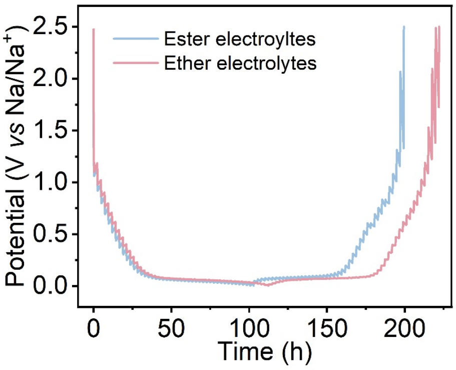

Supplementary Figure 19 | GITT tests of Na||hard carbon half cells. The GITT profiles in 1 M $NaPF_6$-EC/DEC electrolytes and 1 M $NaPF_6$-G2 electrolytes.

The GITT experiment was conducted in a Na||hard carbon half-cell. The cycling protocol consists in 20 mA g^{-1} current pulses for 10 min alternated with 30 min OCV periods.

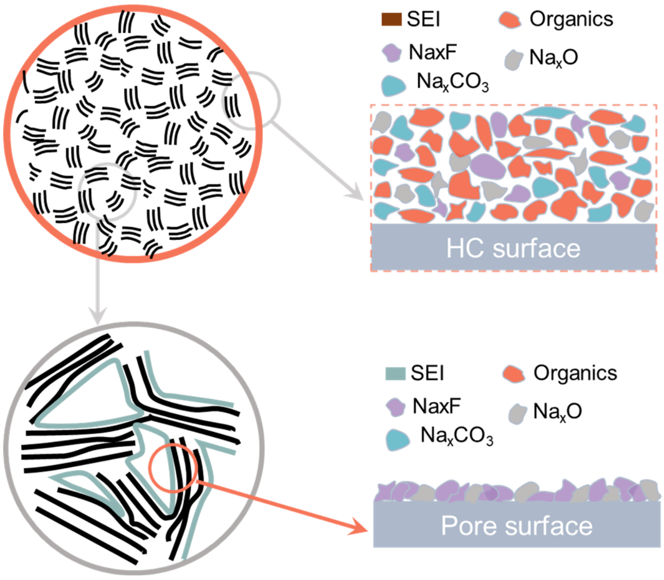

Supplementary Figure 20 | Schematic diagram of SEI formed on surface and nanopore. SEI Schematic diagram of the SEI formed in the surface of hard carbons and the SEI formed in inner pore of hard carbons.

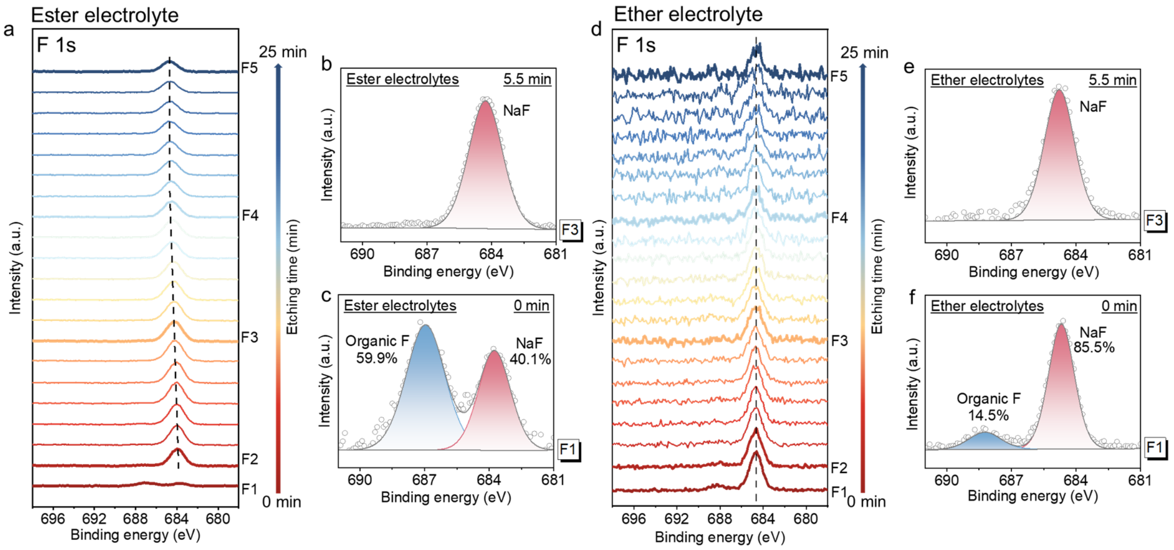

Supplementary Figure 21 | F 1s spectra of hard carbons. a-c, XPS spectra of F 1*s* with different etching time (0-25 min) collected from the cycled hard carbon in 1 M $NaPF_6$-EC/DEC electrolytes (a) and corresponding fitting results at etching time of 0.5 min (b) and 0 min (c). d-e, XPS spectra of F 1*s* with different etching time (0-25 min) collected from the cycled hard carbon in 1 M $NaPF_6$-G2 electrolytes (d) and corresponding fitting results at etching time of 5.5 min (e) and 0 min (f).

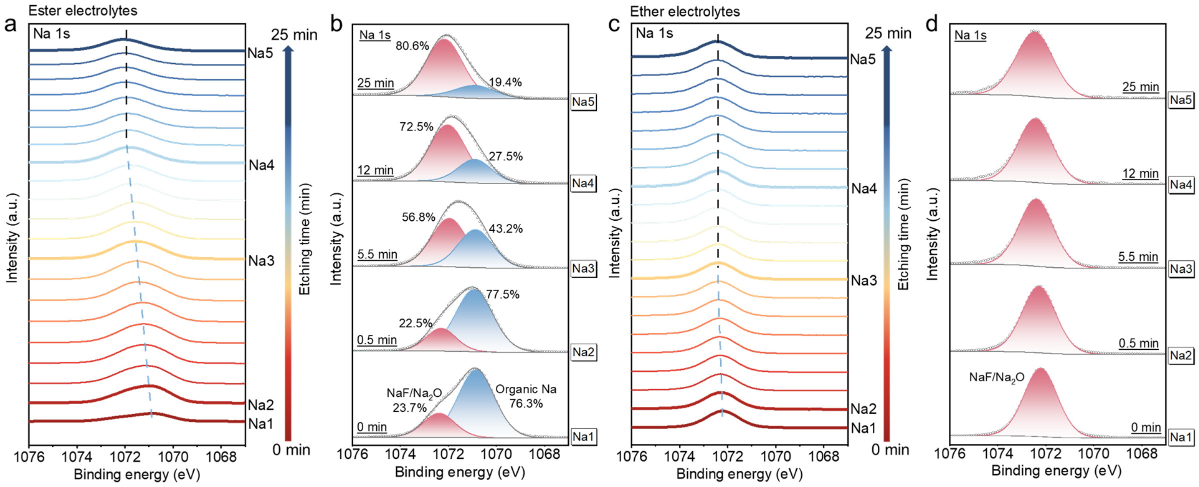

Supplementary Figure 22 | Na 1s spectra of hard carbons. a,b, XPS spectra of Na 1*s* with different etching time (0-25 min) collected from the cycled hard carbon in 1 M $NaPF_6$-EC/DEC electrolytes (a) and corresponding fitting results (b) at etching time of 0, 0.5, 5.5, 12 and 25 min. c,d, XPS spectra of Na 1*s* with different etching time (0-25 min) collected from the cycled hard carbon in 1 M $NaPF_6$-G2 electrolytes (c) and corresponding fitting results (d) at etching time of 0, 0.5, 5.5, 12 and 25 min.

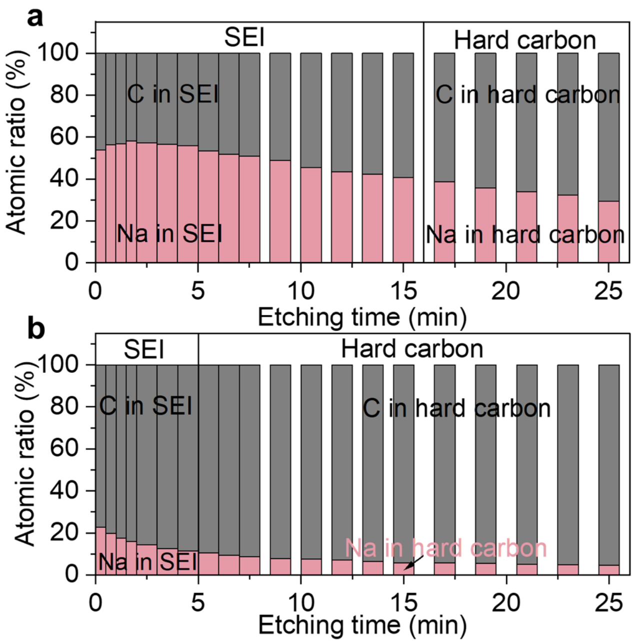

Supplementary Figure 23 | Comparison of relative content of C and Na in cycled hard carbons. The relative proportion of Na and C with etching time from 0-25 min collected from the hard carbon harvested from 1 M $NaPF_6$-EC/DEC electrolytes (a) and 1 M $NaPF_6$-G2 electrolytes (b).

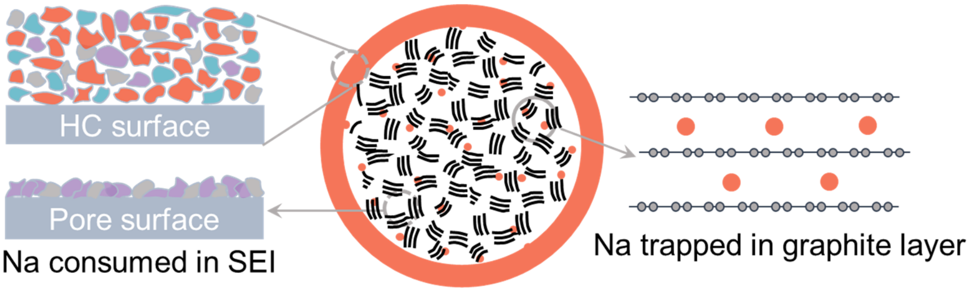

Supplementary Figure 24 | Schematic explanation of irreversible Na loss. Schematic illustration of CE loss due to Na consumption in SEI and intercalation in graphite layers of hard carbons.

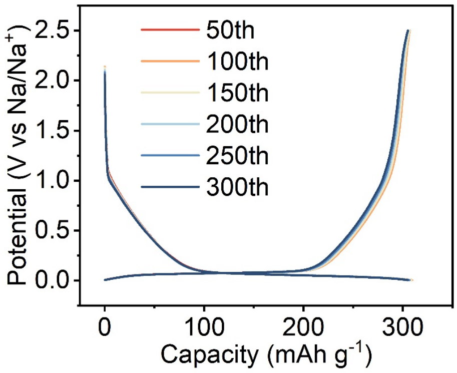

Supplementary Figure 25 | Selected charge-discharge curves of hard carbon anode in 1 M $NaPF_6$-G2 electrolytes. The test was conducted at specific current of 50 mA g^{-1}.

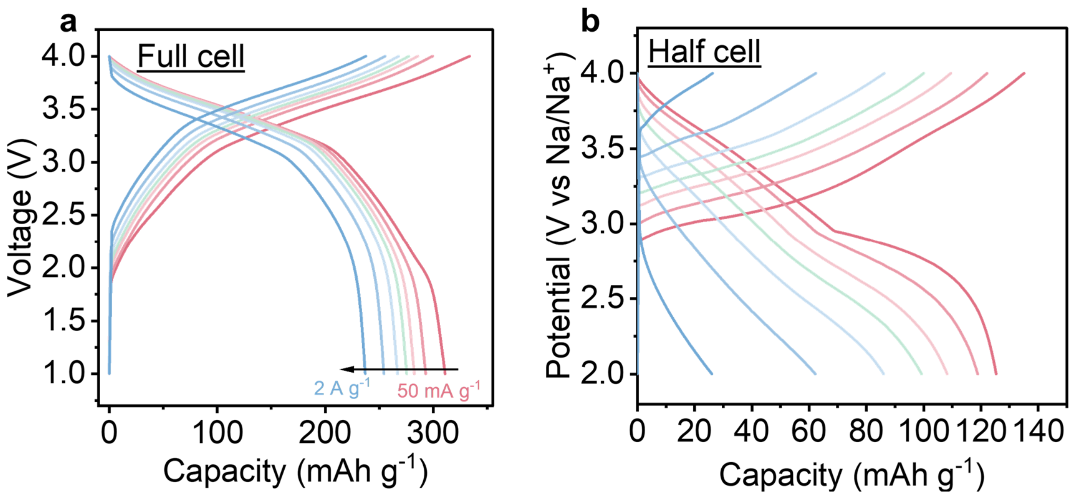

Supplementary Figure 26 | Selected charge-discharge curves. (a) Charge and discharge profiles of full cells at different specific currents (based on the mass of hard carbon anode). (b) Charge and discharge profiles of half cells at different specific currents (based on the mass of NNCFM cathode).

The Na-ion full cell was assembled using hard carbon as the anode and O3 $NaCu_{1/9}Ni_{2/9}Fe_{1/3}Mn_{1/3}O_2$ as the cathode. The weight ratio of anode to cathode was 1:2.42 (negative/positive capacity ratio, N/P ratio = 1.02). For the half cell, it was assembled using Na metal as the anode and NNCFM as the cathode, and Na metal was in sufficient excess compared to the NNCFM electrode.

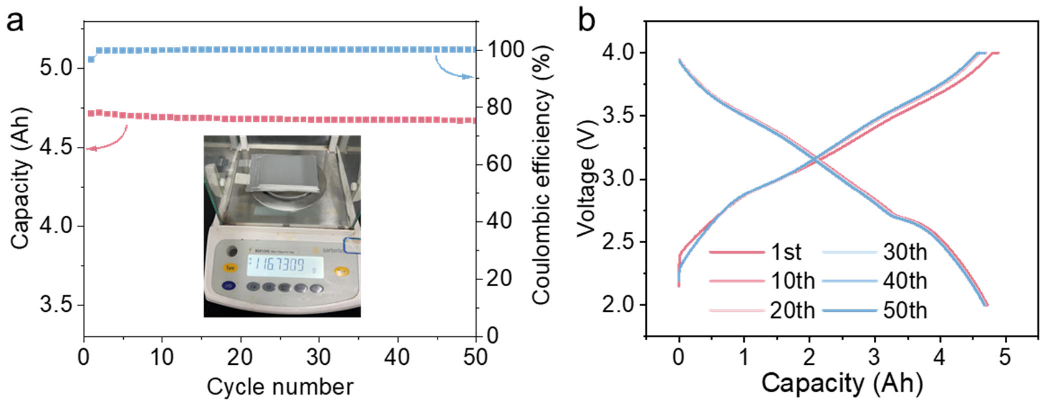

Supplementary Figure 27 | Pouch cell performance. (a) Cycling performance of pouch cell. (b) Selected charge-discharge curves.

The constant current/constant voltage (CC/CV) charging method was used for evaluating the performance of pouch cells. A specific current of 0.5C was applied for the CC charging process, and a cut-off specific current of 1C was used for CV charging process. The discharge process maintains a constant specific current of 0.5C.

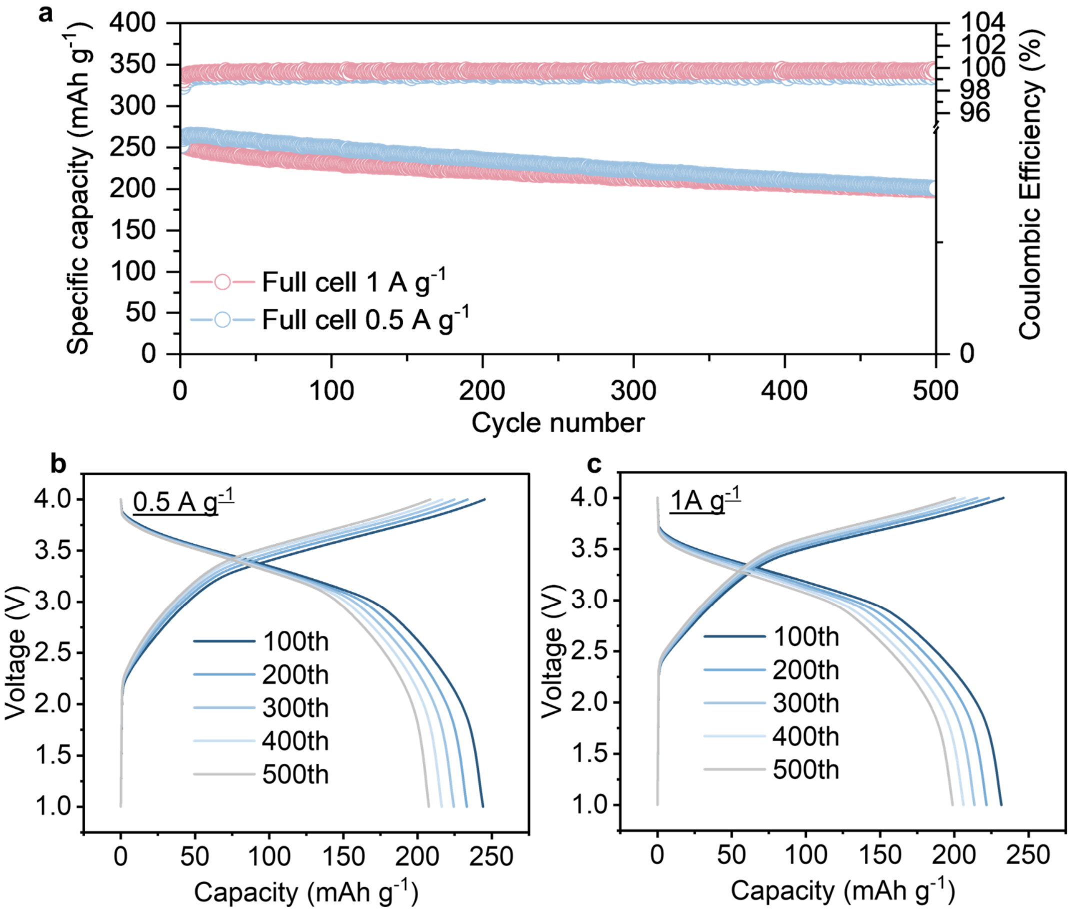

Supplementary Figure 28 | Selected charge-discharge curves of full cells. a, Cycling stability of hard carbon||NNCFM full cell (with unnormalized capacities). Charge and discharge profiles of full cells at specific currents of 0.5 A g^{-1} (b) and 1 A g^{-1} (c).

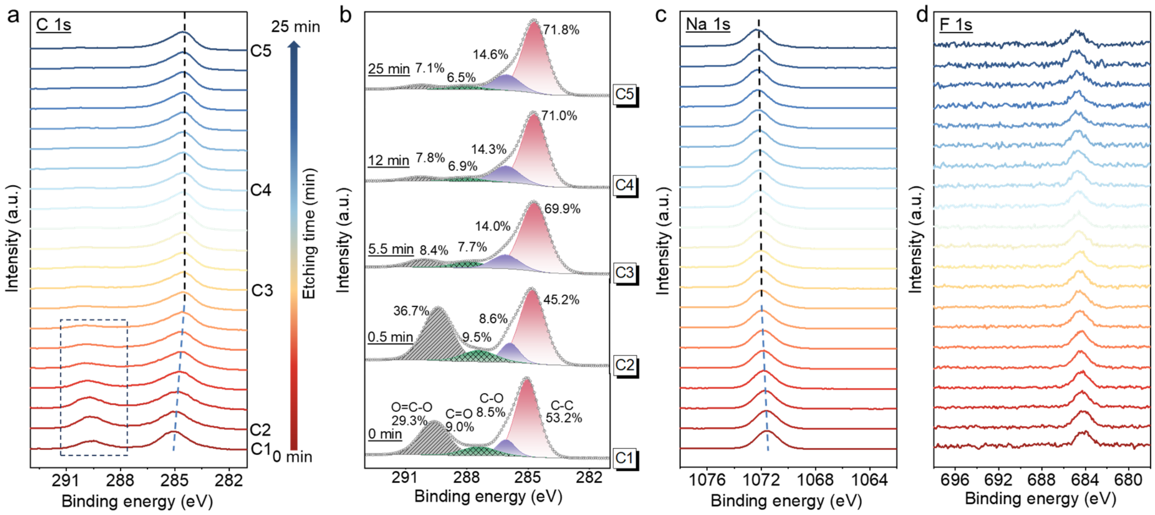

Supplementary Figure 29 | XPS spectra of hard carbons after 500 cycles in full cells. a,b, XPS spectra of C 1*s* with different etching time (0-25 min) collected from the cycled hard carbon in 1 M $NaPF_6$-G2 electrolytes (a) and corresponding fitting results (b) at etching time of 0, 0.5, 5.5, 12 and 25 min. c,d, The Na 1*s* (c) and F 1*s* (d) spectra with different etching time (0-21 min).

With etching, the C 1*s* and Na 1*s* peaks shift towards high and low binding energies, respectively due to the formation of S-SEIs containing organic components with electron-withdrawing properties. After etching for 3.5 min, the C 1*s* and Na 1*s* peak reach stabilization simultaneously. At the same time, organic-related peaks are basically disappeared, suggesting that the S-SEI is completely removed. The thickness of S-SEI is basically unchanged since the S-SEI also disappears after 3.5 min etching for the hard carbon after 500 cycles. However, the composition of S-SEI has changed significantly. The content of O=C-O species on the surface of S-SEI (after 10 cycles) is 11.2% and it becomes 8.4% after etching for 0.5 min. But for the S-SEI formed on the hard carbon after 500 cycles, the proportion of O=C-O species on the surface is 29.3%, which increases to 36.7% after etching for 0.5 min. The content of organic components in I-SEI can also see an increase compared to that after 10 cycles. The proportion of O=C-O species in the I-SEI increases from about 5% to more than 7%. Inorganic components are usually represented by NaF,

and its distribution and content can be inferred from the F 1s spectra. The F 1s peak position is basically unchanged, only the intensity can see a slight decrease with etching, suggesting that the distribution of F-related organic species in the S-SEI and I-SEI is relatively uniform.

Supplementary table 1. Comparison of ICE obtained under corresponding test conditions.

ICE	The type of hard carbons	Electrolyte	Binder	Separator	Specific current	Refs.
98.21%	HC Microspheres (Carbonizing glucose-based precursor at 1400 °C for 2h)	1 M $NaPF_6$-G2	SA	PP	20 mA g^{-1}	This work
77.63%	HC microspheres (Carbonizing 3-aminophenol -based precursor at 1300 °C for 4h with ZnO-assisted bulk etching)	1 M $NaPF_6$-G2	PVDF	Glass fiber	50 mA g^{-1}	1
84%	Zn doping HC (Carbonizing 2, 4-diaminophenol-based precursor and zinc acetate dihydrate at 1300 °C for 2h)	1 M $NaPF_6$-G2	PVDF	Glass fiber	50 mA g^{-1}	2
83.8%	HC microspheres (Carbonizing sucrose-based precursor at 1000 °C for 2h)	0.8 M $NaPF_6$-G2	SA	PP	20 mA g^{-1}	3
85.9%	HC granular (Carbonizing chitosan at 1100 °C for 2h)	1 M $NaPF_6$-DME	SA	PP	50 mA g^{-1}	4
92.1%	Commercial HC (Provided by Kureha)	1 M $NaClO_4$-G4	PVDF	PP	50 mA g^{-1}	5
93%	Carbon nanofiber (Carbonizing fermentation at 1300 °C for 6h)	1 M NaOTf-G2	-	Glass fiber	200 mA g^{-1}	6
84.93%	HC nanospheres (Carbonizing xylose-based precursor at 1200 °C for 3h)	1 M $NaClO_4$-G2	SA	Glass fiber	1000 mA g^{-1}	7
95.0%	Commercial HC (provided by Kureha)	0.5 M $NaBPh_4$-DME	CMC	Glass fiber	20 mA g^{-1}	8
91.2%	HC paper (Carbonizing tissue at 1300 °C for 3h)	1 M NaOTf-G2	CMC	-	20 mA g^{-1}	9

Sodium alginate: SA; Carboxymethyl cellulose sodium: CMC; Poly(vinylidene fluoride): PVDF; Polypropylene: PP.

Supplementary table 2. The EIS fitting results of Na||hard carbon half cells recorded at different aging time and corresponding equivalent circuits.

**Time**	$\mathbf{R_0}$	$\mathbf{CPE_1}$	$\mathbf{R_{ct}}$	$\mathbf{W_o}$
4h	3.21	0.85	86.23	67.07
8h	3.11	0.85	38.45	18.26
12h	3.11	0.86	33.42	18.76
20h	3.14	0.84	28.40	19.34
32h	3.35	0.87	23.56	21.31
40h	3.15	0.85	14.64	17.87
48h	3.12	0.80	11.65	15.27
72h	3.06	0.76	10.06	12.36
96h	3.15	0.79	9.50	10.23

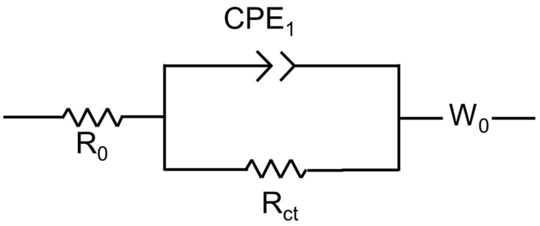

The end of the table shows the equivalent circuit used for fitting EIS data, which consists of four elements, including the internal resistance (R_0), charge transfer resistance (R_{ct}), constant phase element (CPE_1), and the Warburg's element (W_o).

Supplementary table 3. The EIS fitting results of Na||hard carbon half cells at different discharge state in ester electrolytes and corresponding equivalent circuits.

**DOD**	**R_0**	**CPE_1**	**R_{SEI}**	**CPE_2**	**R_{ct}**
OCP	3.07	0.81	171.8	-	-
0.078	3.13	0.84	138.7	0.59	169.0
0.120	2.96	0.86	156.2	0.63	266.3
0.172	3.41	0.87	163.8	0.66	222.0
0.311	4.23	0.86	198.3	0.57	244.7
0.390	3.01	0.87	218.3	0.60	255.3
0.467	3.37	0.88	165.5	0.58	166.4
0.545	2.94	0.90	139.6	0.50	118.2
0.622	2.21	0.89	119.3	0.51	96.2
0.701	2.57	0.87	113.6	0.51	102.7
0.730	2.96	0.90	125.5	0.52	101.3
0.778	2.87	0.90	121.9	0.50	103.4
0.900	2.37	0.89	112.0	0.47	96.6
1.000	5.23	0.89	137.9	0.46	97.7

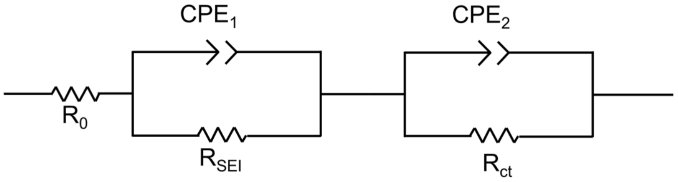

The end of the table shows the equivalent circuit used for fitting EIS data, which consists of four elements, including the internal resistance (R_0), the resistance of the surface films (SEI) of the hard carbon anode (R_{SEI}), and corresponding constant phase element (CPE_1), the charge transfer resistance (R_{ct}), and corresponding constant phase element (CPE_2).

Supplementary table 4. The EIS fitting results of Na||hard carbon half cells at different discharge state in ether electrolytes and corresponding equivalent circuits.

**DOD**	**R_0**	**CPE_1**	**R_{SEI}**	**CPE_2**	**R_{ct}**
OCP	7.81	0.77	40.43	-	-
0.094	6.92	0.81	29.10	0.49	35.37
0.150	11.79	0.79	28.46	0.38	28.68
0.193	9.42	0.82	18.11	0.43	20.92
0.289	10.78	0.86	11.80	0.49	7.58
0.376	11.99	0.89	12.89	0.51	5.51
0.471	10.57	0.84	10.72	0.52	3.71
0.594	11.22	0.86	9.89	0.55	3.64
0.665	10.27	0.85	10.71	0.53	3.63
0.753	10.70	0.84	9.87	0.61	2.98
0.847	7.47	0.88	6.24	0.60	3.57
1.000	11.44	0.90	5.90	0.62	3.37

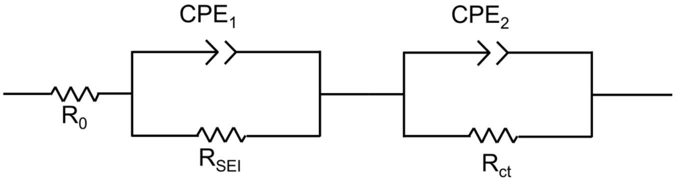

The end of the table shows the equivalent circuit used for fitting EIS data, which consists of four elements, including the internal resistance (R_0), the resistance of the surface films (SEI) of the hard carbon anode (R_{SEI}), and corresponding constant phase element (CPE_1), the charge transfer resistance (R_{ct}), and corresponding constant phase element (CPE_2).

Supplementary References

1 Yin, X. *et al.* Enabling fast Na+ transfer kinetics in the whole-voltage-region of hard-carbon anodes for ultrahigh-rate sodium storage. *Adv. Mater.* **34**, 2109282 (2022).

2 Lu, Z. *et al.* Zinc Single-Atom Regulated Hard Carbons for High Rate and Low Temperature Sodium Ion Batteries. *Adv. Mater.* **45**, 2211461 (2023).

3 Bai, P. *et al.* Long cycle life and high rate sodium-ion chemistry for hard carbon anodes. *Energy Storage Mater.* **13**, 274-282 (2018).

4 He, Y., Bai, P., Gao, S. & Xu, Y. Marriage of an ether-based electrolyte with hard carbon anodes creates superior sodium-ion batteries with high mass loading. *ACS Appl. Mater. Interfaces* **10**, 41380-41388 (2018).

5 Xiao, B. *et al.* Lithium-pretreated hard carbon as high-performance sodium-ion battery anodes. *Adv. Energy Mater.* **8**, 1801441 (2018).

6 Yang, H., Xu, R. & Yu, Y. A facile strategy toward sodium-ion batteries with ultra-long cycle life and high initial Coulombic Efficiency: Free-standing porous carbon nanofiber film derived from bacterial cellulose. *Energy Storage Mater.* **22**, 105-112 (2019).

7 Dong, R. *et al.* Elucidating the mechanism of fast Na storage kinetics in ether electrolytes for hard carbon anodes. *Adv. Mater.* **33**, 2008810 (2021).

8 Morikawa, Y., Yamada, Y., Doi, K., Nishimura, S.-i. & Yamada, A. Reversible and high-rate hard carbon negative electrodes in a fluorine-free sodium-salt electrolyte. *Electrochemistry* **88**, 151-156 (2020).

9 Hou, B. H. *et al.* Self-supporting, flexible, additive-free, and scalable hard carbon paper self-interwoven by 1D microbelts: superb room/low-temperature sodium storage and working mechanism. *Adv. Mater.* **31**, 1903125 (2019).

10. Pré P, Huchet G, Jeulin D, Rouzaud J-N, Sennour M, Thorel A. A new approach to characterize the nanostructure of activated carbons from mathematical morphology applied to high resolution transmission electron microscopy images. *Carbon* **52**, 239-258 (2013).

11. Li J, Ouyang H, Wang J, Li J, Zhang H. Nanostructure Quantification of Hard Carbon Electrodes through Advanced HRTEM Image Analysis. *J. Electrochem. Soc.* **169**, 090522 (2022).

12. Li J, Li T, Peng C, Li J, Zhang H. Molecular structure evaluation and image-guided atomistic representation of hard carbon electrodes. *J. Electrochem. Soc.* **169**, 070517 (2022).
